# Cardiorespiratory Fitness and Muscular Strength Do Not Predict Social Cognitive Capacity in Older Age

**DOI:** 10.1093/geronb/gbad101

**Published:** 2023-07-22

**Authors:** Sarah A Grainger, Julie D Henry, Manikya Alister, Xanthia E Bourdaniotis, Jessica Mead, Tom G Bailey, Jeff S Coombes, Natalie Vear

**Affiliations:** School of Psychology, University of Queensland, St Lucia, Queensland, Australia; School of Psychology, University of Queensland, St Lucia, Queensland, Australia; School of Psychological Sciences, University of Melbourne, Melbourne, Victoria, Australia; School of Psychology, University of Queensland, St Lucia, Queensland, Australia; School of Psychology, University of Queensland, St Lucia, Queensland, Australia; School of Human Movement and Nutrition Sciences, University of Queensland, St Lucia, Queensland, Australia; School of Nursing, Midwifery and Social Work, University of Queensland, St Lucia, Queensland, Australia; School of Human Movement and Nutrition Sciences, University of Queensland, St Lucia, Queensland, Australia; School of Nursing, Midwifery and Social Work, University of Queensland, St Lucia, Queensland, Australia; (Psychological Sciences Section)

**Keywords:** Cardiorespiratory fitness, Cognitive function, Muscular strength, Social cognition

## Abstract

**Objectives:**

Social cognitive function often declines in older age but the mechanisms underlying these declines are not completely clear. Cardiorespiratory fitness (CRF) and muscular strength are positively associated with broader cognitive function in older adults, yet surprisingly, no study has examined whether a similar relationship exists between CRF or muscular strength and social cognition in older age.

**Methods:**

We assessed whether higher CRF and muscular strength were associated with enhanced social cognitive function in a sample of fifty older adults (*M*_age_ = 70.08, standard deviation = 3.93). Participants completed a gold-standard cardiopulmonary exercise test to assess CRF, an isometric handgrip strength test to index muscular strength, and validated measures of social cognition to index emotion perception and theory of mind (ToM).

**Results:**

The results showed that CRF and muscular strength did not explain any unique variance in older adults’ social cognitive performance. Bayesian analyses confirmed that the evidence for the null hypothesis was moderate for all tested relationships, except for the relationship between CRF and cognitive ToM where the evidence for the null was anecdotal.

**Discussion:**

This study has provided the first evidence to suggest that CRF and muscular strength—two important modifiable lifestyle factors—are not associated with social cognition in healthy older adults. However, replication studies are now needed to cross-validate these findings and to clarify whether any moderating variables may be important for understanding the relationship between fitness and social cognition in older age.

Social cognition refers to our ability to detect, understand, and respond to social information. Importantly, it underlies our capacity to engage effectively with others, and therefore has implications for social relationships and broader well-being (Holt-Lunstad et al., 2010; Krendl et al., 2022). An extensive literature now shows that older age is associated with declines in social cognitive function (Grainger et al., 2023a; Henry et al., 2023), with the most robust age effects documented for emotion perception and theory of mind (ToM). Indeed, relative to their younger counterparts, older adults often show difficulties recognizing facial emotions (i.e., emotion perception; Grainger et al., 2017; Hayes et al., 2020; Isaacowitz et al., 2007) and understanding the mental states of others (i.e., ToM, Grainger et al., 2019; Henry et al., 2013; Krendl et al., 2023).

Various mechanisms have been proposed to explain age effects on social cognition, including age-related changes in brain structure and function (see Henry et al., 2023; Ruffman et al., 2008). Indeed, normal aging is associated with weaker functional connectivity between neural networks that support social cognitive processing as well as volumetric losses in key brain regions that are known to underlie social cognition (Hughes et al., 2019; Reas et al., 2020; Vinke et al., 2018). Older age is also associated with vascular changes that have implications for brain health. For instance, white matter hyperintensities are commonly observed in older adults, and have been linked to a decline in social cognition as well as broader cognitive function (de Leeuw et al., 2002; Kynast et al., 2018; Maillard et al., 2012). Although senescence is inevitable, several factors have been shown to be protective against brain aging, including diet, social engagement, sleep, and physical activity (Baranowski et al., 2020; Benedict et al., 2013; Felix et al., 2021; Kaplan et al., 2022; Namsrai et al., 2023). Of particular interest in the present study is the potential role of physical strength and fitness in understanding age-related social-cognitive decline. Cardiorespiratory fitness (CRF) and muscular strength are two indicators of physical health that typically decline with normal aging (Jackson et al., 2009; Sternäng et al., 2015) and have been linked to many different indicators of brain health (Dercon et al., 2021; Duchowny et al., 2022; Erickson et al., 2014; Hayes et al., 2013, 2017; Johnson et al., 2020). Indeed, prior work has shown that greater muscular strength is associated with higher hippocampal volume and reduced white matter hyperintensities in healthy adults (Frith et al., 2020). Similarly, higher aerobic fitness has been linked to increases in hippocampal volume in older adults as well as greater cerebrovascular function (Brown et al., 2010; Erickson et al., 2009). Several studies have now shown that lower CRF and muscular strength are associated with poorer cognitive function in older age (Farrell et al., 2018; Freudenberger et al., 2016; Fritz et al., 2017; Hayes et al., 2016; Sternäng et al., 2016; Zammit et al., 2019). Surprisingly though, no studies have examined whether a similar relationship exists between CRF or muscular strength and social cognition in older age. This is despite the fact that social cognition overlaps considerably with broader cognitive function (Grainger et al., 2023a; Phillips et al., 2011; Rakoczy et al., 2012), and that social cognition is now formally included as one of the six neurocognitive domains alongside executive function and memory in the DSM-5 (Sachdev et al., 2014). The only work to date that has investigated the relationship between CRF and social cognition was by Ludyga et al. (2020) who tested a sample of middle-aged police officers. They found that higher CRF (indexed via estimated V̇O_2MAX_) was associated with greater accuracy on two different facial emotion perception tasks, suggesting that CRF may indeed be important for social cognitive capacity. However, these effects were small in magnitude and focused exclusively on a highly specialized middle-aged cohort (i.e., police officers), therefore it remains to be established whether CRF is also important for social cognitive function in older age—a critical time in adult development where social cognitive abilities undergo notable change. In younger adults, there appears to be a relationship between broader physical activity levels and emotional intelligence. Indeed, Wang et al. (2020) examined whether self-report physical activity levels were associated with trait emotional intelligence in a large sample of college students. They identified a small positive correlation suggesting that being more physically active is associated with better emotional skills. Similar findings were reported by Acebes-Sánchez et al. (2019) who found a significant but weak correlation between leisure time physical activity and trait emotional intelligence in a large sample of students. Together, these findings suggest that being more physically active—which is beneficial for CRF—may be associated with better social cognitive function in younger adulthood.

As noted earlier, the relationship between muscular strength and social cognition in older age is yet to be investigated. However, considering muscular strength has been linked to broader cognitive function in older adults (Duchowny et al., 2022; Frith et al., 2018; Fritz et al., 2017), and because social cognitive function and broader cognition impose demands on some of the same brain regions (Hiser & Koenigs 2018; Wade et al., 2018), it might be expected that muscular strength also predicts social cognitive capacity in old age. Given these gaps in the literature, we aimed to investigate for the first time whether CRF, and muscular strength are related to social cognition in late adulthood, using validated measures that tap into the two social cognitive domains that are most sensitive to age-related decline: emotion perception and ToM. In addition, we aimed to replicate prior studies in this literature that have identified a relationship between both CRF and strength with broader cognition by including a validated measure of processing speed and executive function.

## Hypotheses

We preregistered our study design and hypotheses on the OSF (https://osf.io/7a9q8/?view_only=e9ce925f9de34ebebfd15b75ee60abf4). Given that CRF and muscular strength are associated with positive effects on brain health, we expected both CRF and strength to be positively associated with social cognition. Specifically, we expected V̇O_2PEAK_ and grip strength to be positively associated with ToM (indexed via performance on the Reading the Mind in the Eyes Test [RMET] and false-belief stories) and emotion perception (indexed via the ADFES-BIV). We did not expect any associations with the control stories task as this task does not require social cognitive processing. In line with prior studies that have shown fitness and strength are associated with cognition in old age, we expected higher CRF and muscular strength to be associated with greater processing speed and executive function (i.e., indexed via performance on the Trail Making Test Part A and B).

## Method

### Participants

No study to date has examined CRF or strength in relation to social cognition in older adults. Therefore, we decided to use the effect size reported by Hayes et al. (2016) for our power calculations, who examined the association between CRF and executive function in a sample of older adults. Although social cognition is distinct from executive function, these two neurocognitive domains exhibit some degree of overlap (Grainger et al., 2023a; Phillips et al., 2011; Rakoczy et al., 2012) and show similar patterns of age-related decline across the lifespan (Ferguson et al., 2021; Grainger et al., 2023a). Hayes et al. (2016) reported a moderate-sized association between V̇O_2PEAK_ and cognitive function in older adults (*R*^2^ = 0.17). Therefore, we determined the minimum number of participants required to detect a moderate-sized increase in *R*^2^ (*f*^2^ = 0.17) with 80% power, using a single predictor, in a model where age, sex, and education are entered as control variables in Step 1, followed by the fitness variable (CRF or muscular strength) in Step 2. This revealed that we required a minimum of 49 participants. All participants were recruited from the local Brisbane community and were compensated $80AUD in gift vouchers for their time. Participants were unable to participate if they reported having: (1) a history of cardiovascular or cerebrovascular disease; (2) a diagnosis of diabetes or uncontrolled hypertension, (3) a neurological or psychiatric condition; or (4) if they had an orthopedic condition that would hinder the performance of the exercise. Furthermore, participants were also excluded from participating in the fitness testing if they recorded a high blood pressure reading (>150 mmHg systolic and/or >90 mmHg diastolic) on the day of testing. All participants were screened for abnormal cognitive function using the Mini-Addenbrooke’s Cognitive Examination (M-ACE; Hsieh et al., 2015). We selected the less conservative cutoff of ≤21 for the M-ACE because our primary goal was to ensure that only healthy participants without a clinical disorder (e.g., dementia) were included in the sample. Moreover, the cutoff of ≤25/30 has been shown to have low specificity (Larner, 2015), which means that healthy participants without genuine cognitive impairment could be unnecessarily excluded. Because our study already had several health-related exclusion criteria (e.g., high blood pressure, history of cardiovascular disease, etc.) that restricted who could be involved in the study, we did not want to unnecessarily exclude participants based on a conservative M-ACE cutoff. Therefore, the less conservative cutoff of ≤21/30 was identified as the most appropriate cutoff to use in this study to ensure we could include as many healthy participants as possible in our sample. We ended up recruiting 59 participants in total. Of these, six participants were excluded because they provided a high blood pressure reading on the day of fitness testing, and therefore did not take part in Session 2, two participants failed to complete their fitness testing session due to COVID lockdowns, and one was excluded for scoring below the cutoff on the M-ACE. After these exclusions, the final sample included 50 participants ranging from 60 to 76 years of age.

### Measures

#### Demographic variables

All participants completed a demographic questionnaire that measured the following variables: age, sex, years of education, ethnicity, height, weight, and self-rated health. Participant demographics are shown in Table 1.

**Table 1.** Demographic Characteristics of the Sample (*N* = 50)

Sample characteristics	%	*M* (*SD*)	Range
Age (years)		70.08 (3.93)	60–76
Education (years)		14.75 (3.10)	7–20
BMI (kg/m^2^)		25.88 (3.81)	19.10–37.11
Self-rated health		3.78 (0.74)	2–5
Sex			
Female	56		
Male	44		
Ethnicity			
Caucasian	92		
Asian	6		
Indigenous Australian	2		
M-ACE		28.02 (1.85)	23–30

*Notes*: BMI = body mass index; M-ACE = Mini-Addenbrooke’s cognitive examination.

Self-rated health was measured on a scale of 1–5 (1 = *poor*, 2 = *fair*, 3 = *good*, 4 = *very good*, and 5 = *excellent*).

#### Cardiorespiratory fitness

A cardiopulmonary exercise test was performed to directly measure CRF. Tests were performed on a cycle ergometer, with expired gases measured using a Parvo, TrueOne 2400 (ParvoMedics Inc, Utah, USA) metabolic system. Tests lasted approximately 8–12 min, commencing at a light cycling load and progressing incrementally, with participants maintaining a pedaling frequency of 60–70 revolutions per minute. Heart rate, blood pressure, and rating of perceived exertion were measured throughout the test. All tests were terminated upon participant volitional fatigue. CRF is expressed as peak oxygen consumption relative to body mass (V̇O_2PEAK_).

#### Muscular strength

Muscular strength was assessed in both the dominant and nondominant hand using an Advanced Hand Dynamometer (Tsutsumi Co Ltd, Tokyo, Japan). Participants were asked to stand with their feet shoulder-width apart with the testing upper arm by the side of the body and the elbow flexed at 90°C. They then squeezed the dynamometer maximally for approximately 3 s, with no accessory movements, while being provided with verbal encouragement. This test was performed three times per arm with >10 s rest between tests. Total grip strength was determined by calculating the average of the maximum value from each arm.

#### Social cognition

##### Theory of mind.

—The Reading the Mind in the Eyes Test (RMET, Baron-Cohen et al., 2001) is a well-validated measure of affective ToM. Participants are required to identify how a protagonist is feeling by inspecting isolated pictures of the eye region and selecting the affective state that best describes the eyes from a list of four possible options. The RMET includes 36 trials, and total scores (out of 36) were converted into a percentage accuracy score.

The ToM Stories task (Phillips et al., 2011) is a validated measure of cognitive ToM and measures one’s ability to engage in belief-state reasoning. The task includes brief written vignettes followed by a question that either requires processing of the belief state of the main character (false-belief stories) or understanding of the situation (control stories). An example of a question in this task is: “How likely is it that John will use the South door?”. All responses to the questions were made on a 6-point scale ranging from “definitely not” to “very likely”. Total accuracy scores were converted into percentage accuracy scores.

##### Social perception.

—We used the Amsterdam Dynamic Facial Expression Set—Bath Intensity Variations (ADFES-BIV; Wingenbach et al., 2016), which consists of brief videos of people depicting different emotional displays, including happiness, sadness, anger, fear, disgust, surprise, embarrassment, pride, and contempt (as well as a neutral display) at three different intensities (low, moderate, and high). We used a combination of low and high-intensity expressions. To reduce the total number of trials in the task, we omitted surprised and neutral videos. We included a total of 96 videos in the task (eight different emotions, 12 trials per emotion) and participants were required to watch each video clip and then select the emotional label that best described the expression from a list of the eight possible emotions. We then created a total accuracy score, which was then converted into a percentage.

#### Cognition

In our preregistration document, we originally planned to examine executive function indexed via phonemic verbal fluency and Stroop interference, and memory indexed via immediate and delayed verbal learning. However, testing was completed during the Coronavirus Disease-2019 (COVID-19) pandemic where mask wearing and social distancing were mandatory. These COVID restrictions made tasks with extensive verbal instructions (e.g., listening to long lists of words) and responses more challenging for older adults to complete. Therefore, we decided not to include data from these tasks and instead used data from the Trail Making Test Part A and B to index processing speed and executive function (as these did not require extensive verbal instructions or responses).

##### Trail Making Test A and B.

—The Trail Making Test Part A and B were used to measure processing speed and executive function, respectively. Standardized instructions were used to administer both tasks. Part A requires participants to draw a line to connect 25 numbers (i.e., 1–25) consecutively as quickly and as accurately as possible. Part B requires participants to draw a line connecting numbers and letters in an alternating fashion, (i.e., 1-A-2-B-3-C, etc.) as quickly and as accurately as possible. Both tasks were completed using pen and paper, and participants were scored by measuring the total time to complete the task in seconds. Lower scores indicated better performance on both tasks.

### Procedure

Participants completed all assessments across two separate testing sessions. At Session 1, participants were asked to provide written informed consent. Next, they completed the brief cognitive screen (M-ACE), after which their blood pressure was measured (in both arms) to ensure they were eligible for the fitness testing in Session 2. After these screening tests, participants were seated in front of a laptop computer and were asked to answer basic demographic questions and then completed the social cognitive and cognitive tasks listed above in one of two counterbalanced orders. Instructions were provided at the start of each task and participants were given opportunities to ask questions if any instruction was unclear. Participants completed additional cognitive and social cognitive tasks, but these were not the focus of this study so are not reported here. All participants were offered brief breaks throughout the testing session, which took approximately two hours to complete. At the end of the first session, participants arranged a time and date to complete their second session, which was always completed within one month of Session 1. In Session 2, participants’ blood pressure was recorded to ensure it was safe for them to participate in the fitness testing. Next, they provided a blood sample and vascular assessments (as part of a separate study), and then completed the muscular strength test followed by the cardiopulmonary exercise test. At the completion of these tasks, they were debriefed and compensated for their time.

### Analytic Approach

As specified in our preregistration document, we used a series of hierarchical multiple-regression analyses to test our hypotheses. In all of these analyses, the control variables (age, sex, and years of education) were entered at Step 1 and the fitness variable (CRF or muscular strength) was entered at Step 2, for each of the outcome variables (RMET, ToM Stories, Control Stories, ADFES-BIV, and Trail Making A and B). We also ran the regression analyses involving CRF with BMI included as an additional control variable at Step 1 (see Supplementary Material). For the cognitive ToM task, we also ran the analyses with the control stories included as a control variable in the analysis and these are reported in Supplementary Material. We made the decision post hoc to follow up our frequentist analyses with Bayesian analyses to confirm the strength of the evidence in favor of the null hypothesis. We compared the Bayesian Information Criterion (BIC) values of a null model (predictors: age, sex, and education) to alternative models that included the additional predictor (either CRF or muscular strength). In line with previously reported conventions (van Doorn et al., 2021), a Bayes Factor (BF_10_) less than 1 was indicative of evidence for the null hypothesis, with a BF_10_ between 0.01 and 0.33 indicative of moderate evidence for the null hypothesis and a BF_10_ between 0.33 and 1 indicative of anecdotal evidence for the null hypothesis.

## Results

### Descriptive Statistics

The descriptive statistics (*M*, *SD*, and range) for the fitness (i.e., CRF and muscular strength), social cognition, and cognition measures are shown in Table 2.

**Table 2.** Descriptive Statistics for the Fitness, Strength, Social Cognition, and Cognition Measures

		Mean (*SD*)			Range		
	*n*	Full sample	Males	Females	Full sample	Males	Females
Relative V̇O_2PEAK_	48	22.59 (7.53)	26.96 (8.68)	18.89 (3.52)	13.30–46.30	14–46.30	13.30–28.9
Handgrip strength (kg)	50	30.92 (9.10)	38.99 (6.73)	24.58 (4.50)	17–51.25	26–51.25	17–35.75
ToM stories (%)	49	76.35 (13.57)	75.00 (12.20)	77.45 (14.73)	50–100	50–100	50–100
Control stories (%)	48	82.44 (15.15)	81.55 (12.88)	83.13 (16.91)	37.50–100	62.50–100	37.50–100
RMET (%)	48	75.23 (9.50)	74.47 (9.20)	75.82 (9.86)	50–91.67	50–88.89	55.56–91.67
ADFES-BIV (%)	48	61.98 (11.22)	57.29 (12.94)	65.33 (8.57)	26.04–82.29	26.04–82.29	51.04–82.29
Trail Making Test A	50	32.41 (9.44)	35.24 (11.11)	30.18 (7.35)	21.00–64.04	22.40–64.04	21.00–47.60
Trail Making Test B	49	73.65 (27.11)	73.83 (27.27)	73.51 (27.48)	27.03–157.21	27.03–157.21	39.13–137.60

*Note*: ADFES-BIV = Amsterdam dynamic facial expression set-bath intensity variations; RMET = reading the mind in the eyes test.

### CRF and Social Cognition

As shown in Table 3, the control variables (age, sex, and education) entered at Step 1, explained 20% of the variance in social perception (i.e., ADFES-BIV) scores. However, adding CRF to the model at Step 2 did not account for additional significant variance. For affective ToM (i.e., RMET), the control variables did not account for significant variance in ToM scores, nor did the inclusion of CRF at Step 2. For cognitive ToM (i.e., ToM Stories), the control variables did not account for significant variance in ToM scores. Adding CRF at Step 2 explained an additional 8% of the variance, but this did not reach significance. For the control task, the control variables at Step 1 and entering CRF at Step 2 explained no variance in task scores. For the Bayesian analyses, it can be seen in Table 4 that all of the alternative models (i.e., where CRF was included) provided evidence for the null hypothesis, with moderate evidence identified for social perception, affective ToM, and the control task; and anecdotal evidence identified for cognitive ToM.

**Table 3.** Hierarchical Multiple Regression Analyses Examining the Association Between Cardiorespiratory Fitness (CRF) and Social Cognition, Controlling for Age, Sex, and Education

		Social perception (*n* = 46)		Affective ToM (*n* = 46)		Cognitive ToM (*n* = 47)		Control task (*n* = 46)		Trail A (*n* = 48)		Trail B (*n* = 47)	
		B	*t* (*p*)	B	*t* (*p*)	B	*t* (*p*)	B	*t* (*p*)	B	*t* (*p*)	B	*t* (*p*)
Step 1	Age	−0.56	1.34 (.188)	−0.50	1.39 (.172)	−0.07	.12 (.903)	0.86	1.49 (.143)	0.40	.12 (.902)	−0.05	.05 (.960)
	Sex	−8.85	2.55 (.014)	−0.88	0.28 (.780)	−1.26	0.28 (.779)	−5.83	1.22 (.228)	8.06	2.89 (.006)	2.67	.29 (.772)
	Education	0.47	0.87 (.392)	0.22	0.45 (.655)	−0.59	0.80 (.426)	1.23	1.57 (.124)	−1.00	2.23 (.031)	−0.84	.57 (.570)
	*ΔR* ^*2*^ *F* (*p*)	0.20, 3.39 (.027)		0.05, 0.74 (.532)		0.02, 0.36 (.783)		0.10, 1.58 (.208)		0.19, 3.37 (.027)		0.01, 0.11 (.953)	
Step 2	CRF	0.18	0.62 (.538)	0.19	0.742 (.462)	0.68	1.88 (.067)	−0.07	0.17 (.869)	−0.16, 0.68 (.498)		0.41, 0.53 (.598)	
	*ΔR* ^*2*^ *F* (*p*)	0.01, 0.39 (.538)		0.01, 0.55 (.462)		0.08, 3.54 (.067)		0.00, 0.03 (.869)		0.01, 0.47 (.498)		0.01, 0.28 (.598)	
	*R* ^*2*^ *F* (*p*)	0.20, 2.60 (.050)		0.06, 0.69 (.604)		0.10	1.17 (.338)	0.10, 1.17 (.340)		0.20, 2.61 (.049)		0.01, 0.15 (.960)	

**Table 4.** Bayes Factors (BF_10_) Comparing Evidence for CRF and Strength Models Against the Null

Outcome	Predictor	
	CRF	Strength
Social perception	0.183	0.147
Affective ToM	0.200	0.195
Cognitive ToM	0.976	0.235
Control task	0.150	0.146
Trail making A	0.187	0.142
Trail making B	0.171	0.996

*Note*: Bayes factors were obtained using the “bayestestR” package (version 0.13.0) in R (version 4.2.2) by comparing the Bayesian Information Criterion (BIC) values of a null model (predictors: sex, education, and age) to alternative models that included the additional predictors of fitness and strength respectively.

### CRF and Cognition

The control variables accounted for 19% of the variance in processing speed scores (i.e., Trail Making A). Adding CRF to the model accounted for an additional 1% of the variance but this did not reach significance. For executive function (i.e., Trail Making B), neither the control variables, or CRF accounted significant variance in test scores (see Table 3). The Bayesian analyses showed that the alternative models (i.e., with CRF included in the model) provided moderate evidence for the null hypothesis for both processing speed and executive function.

### Muscular Strength and Social Cognition

Table 5 shows that the control variables accounted for 19% of the variance in social perception (i.e., ADFES-BIV) scores but adding muscular strength at Step 2 failed to explain any additional variance. For affective and cognitive ToM, as well as the control task, the control variables did not explain any significant variance in task performance, and adding muscular strength at Step 2 failed to explain any further variance (see Table 5). For the Bayesian analyses, as shown in Table 4 that the alternative models provided moderate evidence for the null hypothesis for all three social cognitive tasks as well as the control task.

**Table 5.** Hierarchical Multiple Regression Analyses Examining the Association Between Strength and Social Cognition, Controlling for Age, Sex, and Education

		Social perception (*n* = 48)		Affective ToM (*n* = 48)		Cognitive ToM (*n* = 49)		Control task (*n* = 48)		Trail A (*n* = 50)		Trail B (*n* = 49)	
		B	*t* (*p*)	B	*t* (*p*)	B	*t* (*p*)	B	*t* (*p*)	B	*t* (*p*)	B	*t* (*p*)
Step 1	Age	−0.61	1.49 (.144)	−0.15	1.18 (.243)	−0.04	.08 (.941)	0.87	1.51 (.137)	0.04	.12 (.905)	−0.03	.03 (.976)
	Sex	−8.32	2.46 (.018)	−0.37	0.33 (.742)	−0.75	0.17 (.864)	−4.91	1.04 (.305)	7.25	2.56 (.014)	2.48	.28 (.780)
	Education	0.52	0.98 (.331)	0.02	0.13 (.897)	−0.71	1.00 (.324)	1.10	1.43 (.161)	−0.91	2.00 (.052)	−0.87	.62 (.538)
	*ΔR* ^*2*^ *F* (*p*)	0.19, 3.35 (.027)		0.04, 0.55 (.651)		0.03, 0.46 (.713)		0.09, 1.40 (.257)		0.15, 2.63 (.061)		0.01, 0.13 (.942)	
Step 2	Strength	−0.14	0.45 (.656)	0.08	0.74 (.464)	−0.37	0.95 (.348)	0.06	0.15 (.883)	0.02, 0.07 (.947)		1.46, 1.90 (.063)	
	*ΔR* ^*2*^ *F* (*p*)	0.00, 0.20 (.656)		0.01, 0.55 (.464)		0.02, 0.90 (.348)		0.00, 0.02 (.883)		0.00, 0.00 (.947)		0.08, 3.63 (.063)	
	*R* ^*2*^ *F* (*p*)	0.19, 2.52 (.055)		0.05, 0.55 (.704)		0.05, 0.57 (.687)		0.09, 1.03 (.403)		0.15, 1.93 (.121)		0.08, 1.01 (.413)	

### Muscular Strength and Cognition

For processing speed (Trail Making A), the control variables accounted for 15% of the variance in task scores, although this was not significant. Adding muscular strength to the model did not account for any further variance. For executive function (i.e., Trail Making B), the control variables did not account for significant variance in task scores. Muscular strength accounted for 8% of the variance in task scores but this did not reach significance. See Table 5. The Bayesian analyses indicated that the alternative models (i.e., with muscular strength in the model) provided moderate evidence for the null hypothesis for processing speed but only anecdotal evidence for executive function (see Table 4).

## Discussion

It is now well established that aging is associated with declines in core aspects of social cognitive function (Grainger et al., 2023a; Henry et al., 2023), but less well understood are the potential factors that might be protective against such declines. Here, we provided the first test of whether two key indicators of physical functioning—CRF and muscular strength—are associated with social cognitive function in older age. In contrast to our hypotheses, the results showed that CRF and muscular strength did not account for significant variance in older adults’ social cognitive abilities, after controlling for age, sex, and years of education. Bayesian analyses confirmed that the evidence for these null findings was moderate for all social cognitive tasks, with the only exception being between CRF and cognitive ToM where the evidence for the null was anecdotal. As noted earlier, although several studies have shown that CRF and muscular strength predict broader cognitive function in older age (Farrell et al., 2018; Freudenberger et al., 2016; Fritz et al., 2017; Hayes et al., 2016; Sternäng et al., 2016; Zammit et al., 2019), the research is much more limited with respect to social cognition, with only one study completed to date with middle-aged adults. In this study, Ludyga et al. (2020) provided evidence to suggest that fitness may be important for social cognitive function, by showing that CRF estimated from a submaximal exercise test was associated with performance on two separate emotion perception tasks in a sample of police officers. However, the actual proportion of variance accounted for by CRF in their study was small (only 2% in two of their emotion perception tasks). Moreover, CRF was unrelated to performance on their third emotion perception task, which suggests that any benefit of fitness on social cognition may not generalize to all task types. It is also worth noting that Ludyga et al. used an *estimated* measure of CRF rather than a direct measure, therefore the next important step will be to replicate their findings with CRF measured directly via a cardiopulmonary exercise test. In our study, CRF was measured directly using a cardiopulmonary exercise test but it did not predict emotion perception abilities in our older adult cohort, accounting for less than 1% of the total variance in emotion perception scores. However, CRF did account for approximately 8% of the variance in cognitive ToM scores, although this did not reach statistical significance (and Bayesian analyses failed to provide conclusive evidence for this effect). Our findings also do not align with prior work showing that broader physical activity levels are associated with emotional skills in young adulthood (Acebes-Sánchez et al., 2019; Wang et al., 2020). However, consistent with Ludgya et al. (2020), in both of these studies, the relationships identified between physical activity and emotional intelligence were only small in magnitude. Furthermore, these prior studies relied on self-report measures for both physical activity and emotional intelligence, whereas in our study, objective measures were used to assess both CRF and social cognition. Prior work has shown that self-report measures do not correlate strongly with objective measures for physical activity or social cognition (see Grainger et al., 2023b; Prince et al., 2008), therefore differences in measurement type might explain the discrepancy in findings. Indeed, a meta-analysis examining the relationship between emotional intelligence and health reported larger correlations for self-report compared to objective measures of emotional intelligence (Martins et al., 2010). Finally, it is worth noting that Wang et al. (2020) reported a larger variance in task scores compared to our study, which also may have explained why they detected a small effect whereas our study did not. Taken together, these findings suggest that even if some aspects of social cognitive function and CRF are related, the actual strength of these associations appears to be weak and does not appear to generalize across all social cognitive tasks. Our study also aimed to replicate prior work that has shown that both CRF and strength are associated with broader cognitive function. However, in contrast to our predictions, we found no evidence that CRF or muscular strength was associated with processing speed or executive function in our sample. Although inconsistent with most prior work, these findings do align with three recent studies (Silva-Fernandes et al., 2022; Mason et al., 2022; Nilsson et al., 2022) which failed to find a significant association between CRF and cognition after controlling for age, sex, and education. Together, these findings along with our own findings raise the possibility that this literature has experienced a publication bias, such that null effect papers have simply not been published. Another possibility is that findings have been selectively reported, whereby only data from cognitive tasks that have produced significant effects have been published. These recently observed null findings are therefore important to consider because they suggest that the effect of CRF on cognition in older age may not be as robust as first thought.

Given that this was the first investigation of the relationship between fitness and social cognition in older adulthood, and only the second to look at social cognition and fitness more broadly, replication studies are now required. Indeed, Ludgya et al. (2020) is the only study to date to find a significant association between fitness and social cognition, and their study focused on a single social cognitive domain (i.e., social perception) and identified a small effect size that did not generalize to all facial emotion tasks administered. It will therefore be important to replicate and extend this study to establish whether the effect of CRF on social cognition in middle age is truly robust. As is often the case in studies focused on healthy aging where older adults self-select to participate in research, the participants engaged in our study were relatively high functioning. Furthermore, due to the conditions of our ethics approval, we had several exclusion criteria (e.g., a history of cardiovascular disease, uncontrolled hypertension, and diabetes) that only allowed healthy older adults to take part in our study. Therefore, future studies with more diverse older adult samples (e.g., older adults with poorer cardiovascular health and those in more advanced age) would be useful to clarify whether the current findings generalize to all older adults with varying levels of health and well-being. It will also be important to replicate our study with a larger sample size that has sufficient power to detect smaller effect sizes. Although our study design was sufficiently powered to detect a moderate-sized effect, based on prior research that has identified moderate-sized relationships between CRF and broader cognition in older adults (Hayes et al., 2016), it is possible that the relationship between fitness and social cognition is in fact small in magnitude and therefore simply was not detected in our sample. Indeed, the only study to date to measure the relationship between CRF and social cognition reported a small effect in a middle-aged sample, and therefore, it is possible that this relationship is also small in older age.

Our study was also limited in that it only assessed physical fitness and strength in relation to social cognitive skills, when other lifestyle factors (e.g., diet) may also be important. Indeed, Ruffman et al. (2016) found that greater adherence to a Mediterranean diet was associated with stronger ToM in older adults. However, the diet was unrelated to emotion perception scores in their study, which suggests that any potential benefits of a healthy diet may not extend to all social cognitive domains. Surprisingly, despite being published over six years ago, Ruffman et al., is the only study to date that has examined the role of dietary factors in relation to older adults’ social cognitive abilities. Ruffman et al.’s study also relied on a single self-report food frequency questionnaire to measure dietary patterns, and in more recent years the validity of such assessments has been called into question (Ravelli & Schoeller, 2020). Therefore, more studies are now needed to replicate and extend Ruffman et al.’s findings, by using objective and subjective assessments of diet, to clarify the role that dietary factors may play in older adults’ social cognitive function. Moreover, it is possible that fitness and diet in combination might more strongly predict older adults’ social cognitive abilities than they do on their own. Consistent with this idea, a recent intervention study conducted over four years concluded that a combination of exercise training, and a healthy diet may lead to improvements in cognition, but that exercise training or diet alone may have little to no effect (Komulainen et al., 2021). Future studies evaluating the combined effect of fitness and diet on social cognitive aging would be useful moving forward.

To conclude, this study provides the first empirical evidence to suggest that CRF and muscular strength are unrelated to social cognitive abilities in older age. Given this area of investigation is still in its infancy, additional studies are now needed to provide a more nuanced understanding of the specific circumstances in which CRF, and muscular strength might be important, if at all, for social cognitive capacity in older age. Social cognitive abilities play a critical role in maintaining social networks (Krendl et al., 2022), with important implications for broader health and well-being (Holt-Lunstad et al., 2010). Therefore, uncovering the potentially modifiable factors that may contribute to social cognitive aging is critical if we are to develop strategies to promote longevity and well-being in old age.

## Supplementary Material

gbad101_suppl_Supplementary_Material

## Data Availability

All data are available on request by contacting the corresponding author (S. A. Grainger).
