# Supplementary material for: Cardiorespiratory Fitness and Muscular Strength Do Not Predict Social Cognitive Capacity in Older Age
Source: J Gerontol B Psychol Sci Soc Sci. 2023 Jul 22;78(11):1824–33. doi: 10.1093/geronb/gbad101 (PMC10645310; doi:10.1093/geronb/gbad101)
Supplement: gbad101_suppl_Supplementary_Material [file gbad101_suppl_supplementary_material.docx]

**Supplemental Material 1**

During the peer review process, we were asked to include BMI as a control variable in the analyses examining CRF. Therefore, these analyses are reported in Supplementary Table 1 below. Given that performance on the control stories task may have constrained performance on the cognitive ToM task, we were also asked to run the regression analyses with the control stories included as a control variable in the analyses for both CRF and muscular strength. These analyses are reported in Supplemental Tables 2 and 3 below.

**Supplemental Table 1**

*Hierarchical Multiple Regression Examining the Association Between CRF and Social Cognition Controlling for Age, Sex, Education and BMI*

|  |  | Social perception  (*n* = 46) | | Affective ToM  (*n* = 46) | | Cognitive ToM  (*n* = 47) | | Control Stories  (*n* = 46) | | Trail A  (*n* = 48) | | Trail B  (*n* = 47) | |
| --- | --- | --- | --- | --- | --- | --- | --- | --- | --- | --- | --- | --- | --- |
|  |  | B | *t* *(p*) | B | *t* *(p*) | B | *t* *(p*) | B | *t* *(p*) | B | *t* *(p*) | B | *t* *(p*) |
| Step 1 | Age | -.55 | 1.25 (.218) | -.18 | 1.39 (.172) | -.04 | .07 (.947) | .87 | 1.50 (.142) | .09 | .28 (.778) | -.23 | .22 (.828) |
|  | Sex | -9.03 | 2.49 (.017) | -.26 | 0.23 (.821) | -1.88 | 0.41 (.683) | -6.18 | 1.25 (.218) | 7.26 | 2.57 (.014) | 5.34 | .57 (.568) |
|  | Education | .50 | 0.88 (.387) | .07 | 0.39 (.698) | -.50 | 0.67 (.507) | 1.28 | 1.59 (.121) | -.87 | 1.92 (.062) | -1.27 | .86 (.396) |
|  | BMI | -.08 | .19 (.852) | .03 | 0.23 (.820) | -.35 | 0.63 (.531) | -.20 | 0.33 (.744) | -.46 | 1.33 (.191) | 1.55 | 1.38 (.174) |
|  | *ΔR^2^ F* (*p*) | .20, 2.49 (.058) | | .05, 0.56 (.694) | | .03, 0.37 (.832) | | .10, 1.19 (.331) | | .22, 3.01 (.028) | | .05, .57 (.690) | |
| Step 2 | CRF | .17 | 0.59 (.561) | .08 | .81 (.424) | .67 | 1.75 (.087) | -.12 | 0.26 (.797) | -.24, 1.03 (.311) | | .69, .88 (.383) | |
|  | *ΔR^2^ F* (*p*) | .01, 0.34 (.561) | | .02, .65 (.424) | | .07, 3.08 (.087) | | .00, 0.07 (.797) | | .02, 1.05 (.311) | | .02, .78 (.383) | |
|  | *R^2^ F* (*p*) | .20, 2.03 (.095) | | .07, 0.57 (.720) | | .10 | 0.92 (.477) | .11, 0.94 (.465) | | .24, 2.62 (.038) | | .07, .61 (.697) | |

**Supplemental Table 2**

*Hierarchical Multiple Regression Examining the Association Between CRF and Cognitive ToM, While Controlling for Age, Sex, Education and Control Stories.*

|  |  | Cognitive ToM  (*n* = 46) | |
| --- | --- | --- | --- |
|  |  | B | *t*(*p*) |
| Step 1 | Age | -.07 | 0.13 (.898) |
|  | Sex | -1.29 | 0.28 (.776) |
|  | Education | -.59 | 0.80 (.429) |
|  | *ΔR^2^ F* (*p*) | .02, 0.35, (.789) | |
| Step 2 | Control Stories | .22 | 1.50 (.141) |
|  | *ΔR^2^ F* (*p*) | .05, 2.26 (.141) | |
| Step 3 | CRF | .79 | 2.05 (.047) |
|  | *ΔR^2^ F* (*p*) | .09 | 4.20 (.047) |
|  | *R^2^ F* (*p*) | .16, 1.56 (.194) | |

**Supplemental Table 3**

*Hierarchical Multiple Regression Examining the Association Between Strength and Cognitive ToM, While Controlling for Age, Sex, Education and Control Stories.*

|  |  | Cognitive ToM  (*n* = 46) | |
| --- | --- | --- | --- |
|  |  | B | *t*(*p*) |
| Step 1 | Age | -.04 | 0.08 (.934) |
|  | Sex | -.80 | 0.18 (.858) |
|  | Education | -.71 | 0.99 (.327) |
|  | *ΔR^2^ F* (*p*) | .03, 0.45, (.719) | |
| Step 2 | Control Stories | .24 | 1.75 (.087) |
|  | *ΔR^2^ F* (*p*) | .07, 3.08 (.087) | |
| Step 3 | Strength | -.38 | 1.00 (.324) |
|  | *ΔR^2^ F* (*p*) | .02, 1.00 (.324) | |
|  | *R^2^ F* (*p*) | .12, 1.10 (.377) | |
